# Predicting Hepatocellular Carcinoma Risk in Chronic Hepatitis B Patients Receiving Finite Periods of Antiviral Therapy

**DOI:** 10.3390/cancers15133343

**Published:** 2023-06-25

**Authors:** Chih-Lang Lin, Szu-Yuan Wu, Ming-Wei Lai, Chao-Wei Hsu, Wan-Ming Chen, An-Tzu Jao, Cheng-Hung Chien, Ching-Chih Hu, Rong-Nan Chien, Chau-Ting Yeh

**Affiliations:** 1 Liver Research Center, Department of Gastroenterology and Hepatology, Keelung Chang Gung Memorial Hospital, Keelung 204, Taiwancashhung@cgmh.org.tw (C.-H.C.);; 2 Community Medicine Research Center, Keelung Chang Gung Memorial Hospital, Keelung 204, Taiwan; 3 College of Medicine, Chang Gung University, Taoyua 833, Taiwanhsu2406@cgmh.org.tw (C.-W.H.); 4 Graduate Institute of Business Administration, College of Management, Fu Jen Catholic University, New Taipei City 242, Taiwan; richardch9@tmu.edu.tw (S.-Y.W.);; 5 Artificial Intelligence Development Center, Fu Jen Catholic University, New Taipei City 242, Taiwan; 6 Big Data Center, Lo-Hsu Medical Foundation, Lotung Poh-Ai Hospital, Yilan 265, Taiwan; 7 Department of Food Nutrition and Health Biotechnology, College of Medical and Health Science, Asia University, Taichung 413, Taiwan; 8 Division of Radiation Oncology, Lo-Hsu Medical Foundation, Lotung Poh-Ai Hospital, Yilan 265, Taiwan; 9 Department of Healthcare Administration, College of Medical and Health Science, Asia University, Taichung 413, Taiwan; 10 Centers for Regional Anesthesia and Pain Medicine, Taipei Municipal Wan Fang Hospital, Taipei Medical University, Taipei City 242, Taiwan; 11 Liver Research Center, Chang Gung Memorial Hospital, Taoyuan 833, Taiwan; 12 Division of Pediatric Gastroenterology, Department of Pediatrics, Linkou Chang Gung Memorial Hospital, Taoyuan 333, Taiwan

**Keywords:** hepatocellular carcinoma, antiviral therapy, hepatitis B virus, predictive scoring, nucleos(t)ide analogue

## Abstract

**Simple Summary:**

Hepatocellular carcinoma (HCC) is a severe complication of chronic hepatitis B virus (HBV) infection. HCC can still develop in CHB patients undergoing antiviral therapy. Although several scoring systems for the prediction of HCC risk in CHB patients are available, very few of them are established for CHB patients receiving antiviral therapy. Our study developed a predictive scoring system for CHB patients on finite antiviral treatment, categorizing them into three risk groups based on a multivariate Cox proportional hazards model. Significant differences in HCC incidence were observed among these groups.

**Abstract:**

PURPOSE: Hepatocellular carcinoma (HCC) is one of the most severe complications in chronic hepatitis B virus (HBV) infection. HCC can still develop in patients with chronic HBV (CHB) infection undergoing antiviral therapy. Several effective scoring systems for the prediction of HCC risk in CHB patients have been established. However, very few of them are designed for CHB patients receiving nucleos(t)ide analogues (NAs) therapy. Furthermore, none are available for HCC risk prediction in CHB patients receiving finite periods of antiviral therapy. METHODS: This study enrolled 790 consecutive treatment-naïve patients with CHB infection who had visited our liver clinics from 2008 to 2012 for pretreatment assessment before receiving antiviral therapies. The treatments were provided at finite periods according to the National Health Insurance Policy in Taiwan. The last follow-up date was 31 December 2021. We analyzed the virological and clinical factors in these 790 CHB patients receiving finite periods of NA treatments and identified the most significant risk factors for HCC to establish a novel predictive scoring system. By using stepwise selection in a multivariate Cox proportional hazards model, we divided the patients into three risk groups. RESULTS: Our predictive scoring system included five independent variables: genotype C (adjusted HR [aHR] = 2.23), NA-withdraw-related hepatitis relapse (aHR = 6.96), male (aHR = 4.19), liver cirrhosis (aHR = 11.14), and T1768A core promoter mutation (aHR = 3.21). This model revealed significant differences in HCC incidence among the three risk groups. The 5-year cumulative HCC risk significantly differed among the three risk groups (low risk: 1.33%, moderate risk: 4.99%, and high risk: 17.46%), with log-rank test *p* < 0.001. CONCLUSION: Our predictive scoring system is a promising tool for the prediction of HCC in CHB patients receiving finite NA treatments. Genotype C, NA-withdraw-related hepatitis relapse, male gender, liver cirrhosis, and the T1768A HBV core promoter mutation were significant independent risk factors.

## 1. Introduction

Hepatocellular carcinoma (HCC), a severe complication of chronic hepatitis B virus (HBV) infection, is a leading cause of cancer-related mortality in Taiwan [1,2,3]. Hepatocellular carcinoma (HCC) risk factors in patients with chronic HBV infection (CHB) encompass a range of factors. These include liver cirrhosis, advanced age, male gender, and various HBV-related factors such as persistent necroinflammation, elevated levels of HBV DNA and HBV surface antigen (HBsAg), infection with HBV genotype C (compared to genotype B), and the presence of specific mutations. Notably, mutations in the basal core promoter region or nonsense mutations in the surface gene have been particularly associated with an increased risk of HCC [4,5].

HBV has a high potential for causing cancer, and the process of HBV-related carcinogenesis is complex and involves multiple factors and pathways [6,7]. Various mechanisms contribute to the oncogenic activity of HBV in hepatocellular carcinoma (HCC). These mechanisms include insertional mutagenesis resulting from the integration of HBV DNA into the host genome, heightened genomic instability caused by HBV DNA integration and the direct impact of viral proteins, as well as the disruption of vital cellular functions such as proliferation, apoptosis, and DNA repair [7,8]. Furthermore, chronic active inflammation plays a significant role in enhancing HBV’s oncogenic potential by inducing increased oxidative stress, necrosis, regeneration, angiogenesis, and cellular senescence, all of which collectively promote mutagenesis and carcinogenesis [8]. Studies have shown that HBV DNA integration into the host genome occurs early in the phase of CHB infection and in the early stages of liver carcinogenesis. This may explain why patients with CHB infection who receive antiviral treatment still have a significant risk of developing HCC [8].

The current recommended treatment for CHB is the use of nucleos(t)ide analogues (NA). These antiviral therapies are effective at inhibiting HBV replication and reducing necroinflammation, both of which are associated with an increased risk of HCC in patients with CHB [9]. While it is expected that antiviral therapy will decrease the incidence of HCC in these patients, some studies have shown that HCC can still develop in patients receiving antiviral treatment, though the risk is significantly reduced with the use of NA therapies [7,10].

Several scoring systems have been developed to predict the risk of HCC in CHB patients [11], but very few are specifically designed to predict the risk of HCC in CHB patients receiving NA therapy [12,13]. To the best of our knowledge, there are no established systems for predicting the occurrence of HCC in CHB patients receiving finite periods of antiviral treatment, despite this strategy being included as an option or recommendation in international treatment guidelines [14,15]. The present study aims to create a predictive scoring system for the risk of HCC in treatment-naive patients with CHB infection receiving finite periods of antiviral therapy. Patients with CHB who have high HCC risk scores, including those receiving NAs, should be closely monitored.

## 2. Patients and Methods

### 2.1. Patients

This retrospective cohort study was conducted under the approval of Institutional Review Board, Chang Gung Medical Hospital (Institutional Review Board number 202002057B0). This study enrolled 790 consecutive treatment-naïve patients with CHB infection who visited our liver clinics from 2008 to 2012 for pretreatment assessment before receiving antiviral therapy. The index date was the initial date of receiving antiviral treatments. The last follow-up date was 31 December 2021. The reason we did not include patients enrolled at other dates is that the interval between receiving antiviral therapy and the last follow-up date is an important factor for assessing HCC risk in treatment-naïve CHB patients receiving finite periods of NA treatments. In our study, we specifically enrolled patients with CHB infection who visited our liver clinics from 2008 to 2012 for pretreatment assessment before receiving antiviral therapy. The index date was set as the initial date of receiving antiviral treatments, and the last follow-up date was 31 December 2021. This ensured that the interval between antiviral therapy and the last follow-up date was at least 9 years, allowing us to evaluate the long-term HCC risk in this patient population. In the current cohort, there were no dropouts due to death, relocation, declined follow-up, or transfer to another hospital. All patients enrolled in the study remained in the follow-up until the last recorded date.

Clinical and virological data were retrieved from the medical records of patients and the data bank of our liver research center, respectively. Patients underwent regular follow-ups and treatment in our outpatient clinics. The diagnosis criteria of HCC were the presence of typical HCC features on dynamic computed tomography and dynamic magnetic resonance imaging and on angiography if the tumor size in a cirrhotic liver exceeded 2 cm [16,17]. When the typical features of HCC were not present, or the tumor size was <2 cm, a liver biopsy or aspiration cytology was required for diagnosis [18]. Finite periods of antiviral treatment were provided according to the National Health Insurance Guideline, with the treatment strategies described in detail previously [19].

### 2.2. Clinical and Virological Variables

Demographic characteristics and clinical parameters, including gender, age, HBsAg, HBe antigen (HBeAg), antibody against HBe (anti-HBe), liver cirrhosis status, date of last follow-up, date of HCC diagnosis, and date of mortality, were retrospectively retrieved from medical records. Biochemistry and hemogram analyses were conducted for alpha-fetoprotein, bilirubin, aspartate transaminase (AST), alanine transaminase (ALT), prothrombin time, and platelet count. It is important to note that the assigned points and cut-off values for each variable in the scoring system were determined based on the standard values derived from laboratory data at Chang Gung Memorial Hospital. For example, an alpha-fetoprotein level between 0 ng/mL and 40 ng/mL is considered normal for adults. In our study, liver cirrhosis was confirmed by the hepatologists at Chang Gung Memorial Hospital based on the results of liver ultrasound performed on the index date (start of antiviral treatment). The diagnosis of liver cirrhosis required confirmation through two or more ultrasound examinations and liver biopsy using the Batts–Ludwig scoring system, with a stage equal to or greater than 2 [20]. Only patients with documented liver cirrhosis based on these criteria were classified as having liver cirrhosis in our study. Virological data were retrieved from the data bank of the Liver Research Center, Chang Gung Memorial Hospital (CGMH), and included HBV DNA levels, genotype, basal core promoter (BCP), and precore mutations. The precore mutations included G1719T, G1728A/C, C1730G, A1752G, T1753C/A, A1762T, G1764A, C1766T, T1768A, C1799G, G1896A, and G1899A (Table 1).

### 2.3. Antiviral Treatment Methods

Antiviral therapies were classified as follows: (1) peginterferon in combination with entecavir, (2) entecavir monotherapy subsequent switching to or addition of other NAs, (3) entecavir monotherapy, (4) tenofovir monotherapy switching to or addition of other NAs, (5) initial lamivudine or telbivudine therapy with or without subsequent rescue therapies, and (6) peginterferon monotherapy.

### 2.4. Statistical Analysis

All statistical analyses were performed using SAS for Windows (version 9.4; SAS Institute, Cary, NC, USA). Statistical significance was indicated by *p* < 0.05. Essential demographic characteristics, namely gender and age, were categorized. Patient age was determined at the index date. The variables of interest were demographic characteristics, laboratory data, relapse after NA treatment, times of antiviral treatment, antiviral regimen changes, liver cirrhosis, HBV genotypes, and HBV viral point mutations. The chi-square test was used to compare demographic characteristics, relapse after NA treatment, times of antiviral treatment, antiviral regimen changes, liver cirrhosis, HBV genotypes, and HBV viral point mutations between the HCC and non-HCC groups (Table 1). Relapse after NA treatment was defined as HBV DNA > 2000 IU/mL two times and ALT > upper limit of normal (ULN).

We employed the forward stepwise selection method to identify significant factors for constructing the CGMH HCC Risk Predictive Scoring System in treatment-naïve patients with CHB infection (Table 2). Factors that demonstrated significant predictive value for HCC risk (*p* < 0.05) were selected using a coefficient of >0 or an adjusted hazard ratio (aHR) of >1. These selected factors were incorporated into the predictive scoring system, assigning points based on their respective aHR values. To ensure the model’s validity, a modified forward selection technique was implemented during the stepwise selection process, eliminating non-significant factors. Both forward selection and backward elimination approaches were utilized to evaluate the factors’ contribution as they were added to or removed from the model. It is worth noting that the “minimum F-to-enter” criterion was employed to determine variable inclusion or exclusion. The most favorable model was chosen on the basis of the Akaike information criterion (AIC). The AIC is a statistical measure utilized for model selection and evaluation. In our study, we employed AIC to compare various predictive models and select the most appropriate one for estimating the risk of HCC in treatment-naïve patients. By evaluating the AIC values, we identified the model with the lowest AIC as the best fit for the data, indicating its superior performance compared to other models. Risk factors were determined based on an adjusted hazard ratio (aHR) of ≥1. Each risk factor was assigned a risk point corresponding to the highest integer equal to or lower than its respective aHR in the stepwise regression analysis [21]. Patients were categorized into three risk groups based on their risk scores, with those in the moderate–high-risk groups predicted to have a higher incidence of HCC. Receiver operating characteristic (ROC) curves were generated for each risk score, and the areas under the ROC curves were calculated. The CGMH predictive scoring system’s ability to predict HCC risk was assessed using the Kaplan–Meier method, and differences among the risk groups were evaluated using the log-rank test. Statistical significance was defined as a two-tailed *p* value of <0.05.

## 3. Results

### 3.1. Demographic Characteristics of Treatment-Naïve Patients with CHB Infection Receiving NAs

We compared the demographic characteristics, laboratory data, hepatitis relapse after NA withdrawal, courses of antiviral treatment (repeated periods of finite treatments are allowed for NA-withdraw-related hepatitis relapse in our National Insurance Policy), antiviral treatment strategies, liver cirrhosis, HBV genotype, and HBV viral core promoter and precore mutations, between the non-HCC and HCC groups of treatment-naïve CHB patients receiving finite NA treatments. Of the 790 patients included in this study, 33 (4.2%) had HCC, and 757 did not have HCC. The male-to-female ratios were 70.2:29.9 and 87.9:12.1 (*p* = 0.028) in the HCC and non-HCC groups, respectively. The mean (standard deviation) age was 48.40 (12.84) and 53.46 (8.34) years (*p* = 0.025) in the HCC and non-HCC groups, respectively. The two groups were compared for clinical and virological parameters, namely AST, ALT, platelet count, HBV DNA titers, anti-HBe, relapse after NA withdrawal, antiviral treatment strategies, liver cirrhosis, HBV genotype, and HBV viral core promoter/precore mutations (see Table 1 for results).

### 3.2. Stepwise Selection for HCC after NAs

Table 2 presents all the significant factors identified through the stepwise method applied to the multivariate model for variable selection. Each risk factor was assigned a score based on its hazard ratio (HR). Following stepwise selection in the multivariate Cox proportional hazards model for HCC in treatment-naïve CHB patients receiving finite NA treatments, several factors were determined as significant independent risk factors for HCC. These factors include genotype C (aHR: 2.23, score: 2), relapse after NA withdrawal (aHR: 6.96, score: 7), male gender (aHR: 4.19, score: 4), liver cirrhosis (aHR: 11.14, score: 11), and T1768A mutation (aHR: 3.21, score: 3).

### 3.3. HCC Assessment Using the CGMH HCC Predictive Scoring System

The risk score, known as the CGMH cumulative score, was calculated by accumulating the assigned scores for each risk factor. As the risk scores increased, the proportion of patients with HCC also increased. For example, the distribution of HCC risk based on the risk scores was as follows: a score of 0 corresponded to an HCC risk of 0.00%, a score of 13 corresponded to an HCC risk of 4.88%, and a score of 22 corresponded to an HCC risk of 14.64%. The results of the HCC risk assessment obtained using the CGMH HCC predictive scoring system are presented in Table 3. We categorized patients into low-risk (score ≤ 12), moderate-risk (score = 13–20), and high-risk (score ≥ 21) groups (Table 4).

### 3.4. HCC Assessment Using the PAGE-B Predictive Scoring System

**Figure 1:**
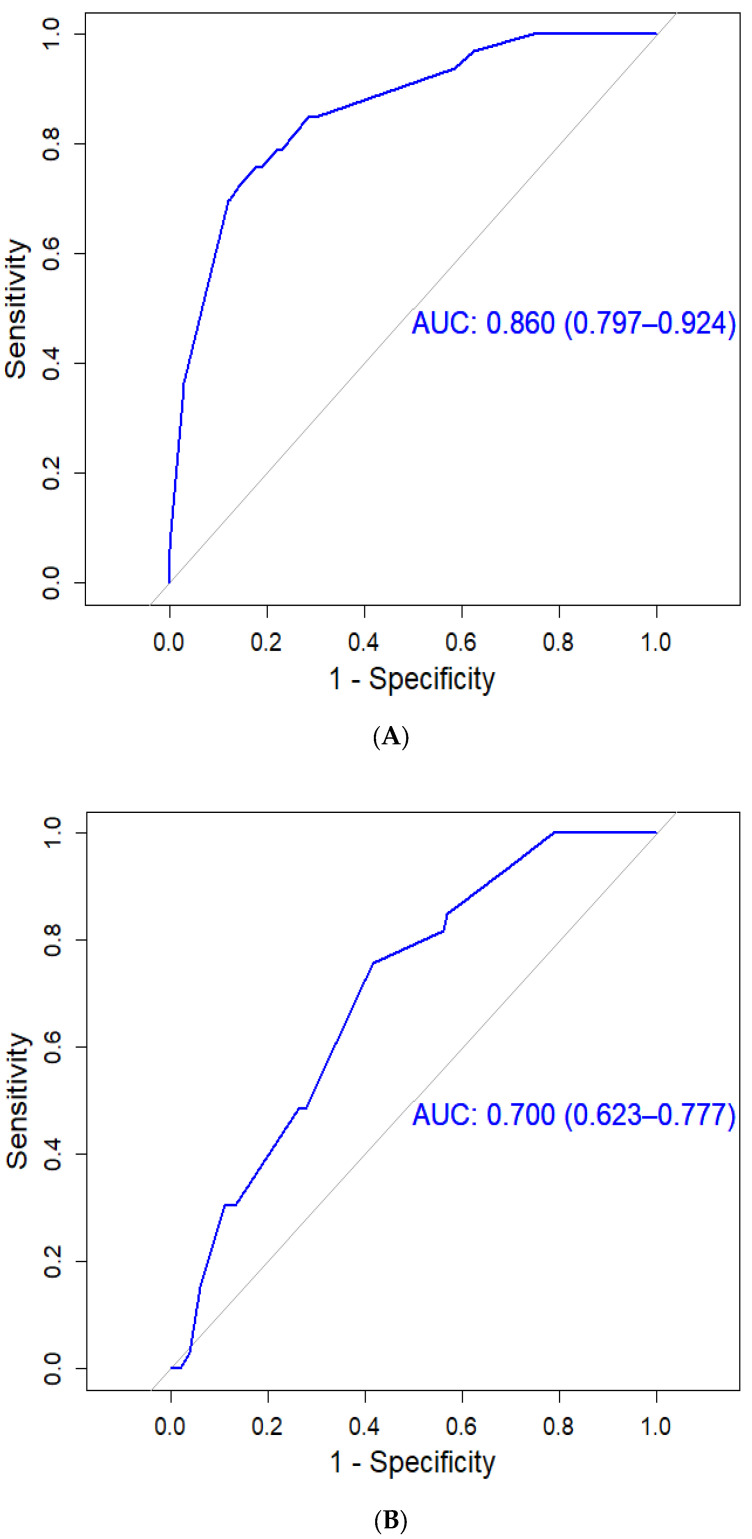
Areas Under the ROC Curves of (**A**) CGMH Predictive and (**A**) PAGE-B Scores CGMH Scores (**B**) PAGE-B Scores.

PAGE-B is another predictive scoring system for predicting HCC risk [22]. PAGE-B is the only other predictive scoring system for HCC risk in patients with CHB infection receiving NAs. PAGE-B was based on the data set including the data of Caucasian patients. Using PAGE-B, the proportion of patients with HCC increased with the accumulation of risk scores (e.g., score, HCC risk [≤9, 0.00%], [10–17, 4.10%], and [≥18, 7.41%]; Supplementary Table S1). The areas under the ROC curves were 0.70 for PAGE-B scores and 0.86 for CGMH scores (Figure 1).

### 3.5. Kaplan–Meier Survival Curve for Cumulative HCC Risk Determined Using the CGMH HCC Predictive Scoring System

**Figure 2:**
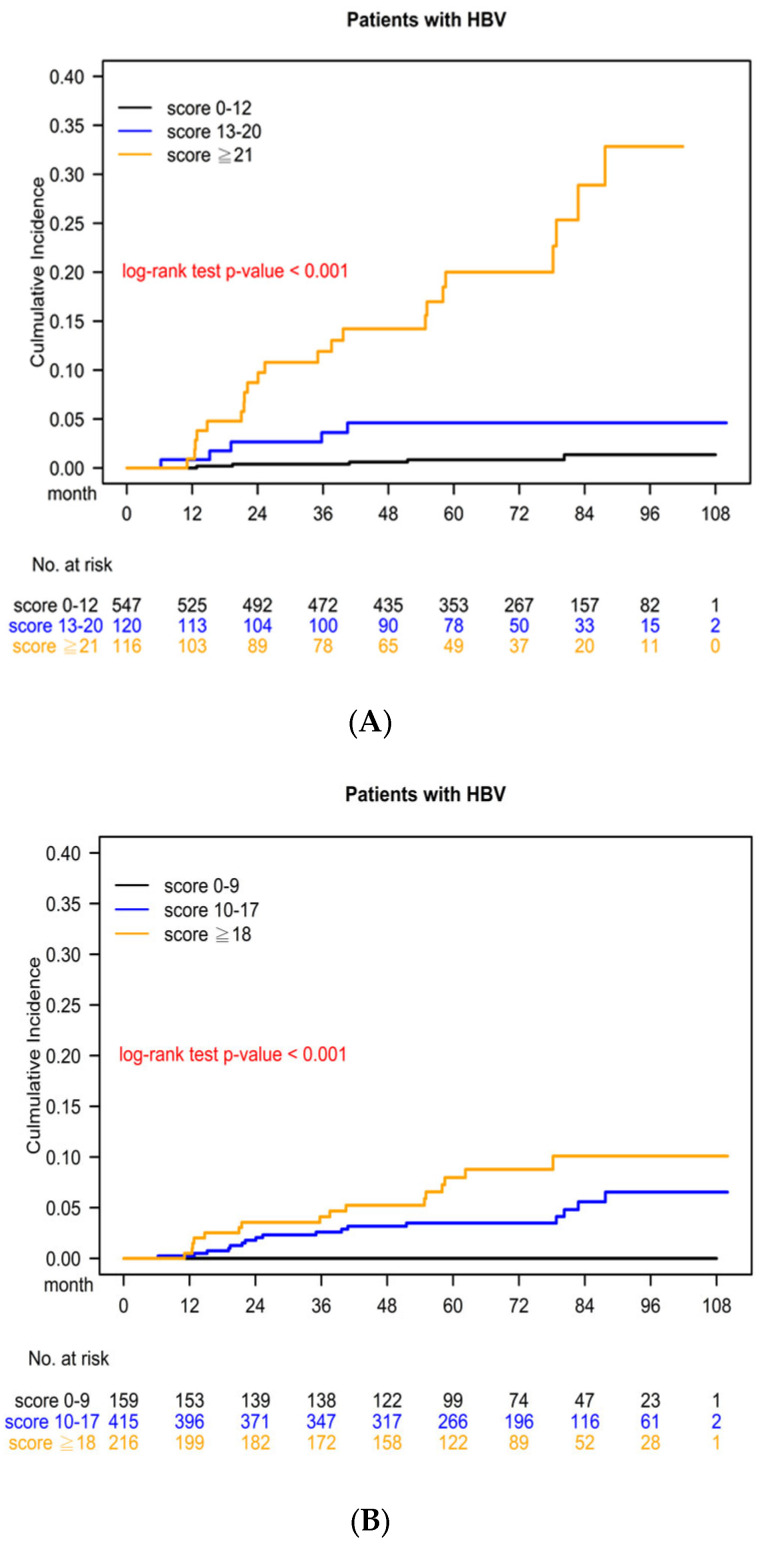
Kaplan–Meier Cumulative Incidence Curve of Hepatocellular Carcinoma for (**A**) CGMH Predictive and (**B**) PAGE-B Scoring Systems in Three Groups. Note: *p* value (log-rank test) < 0.0001 CGMH Scores. (**B**) PAGE-B Scores.

Patients were divided into three groups according to the risk score determined using the CGMH HCC predictive scoring system: low risk (score: 0–12), moderate risk (score: 13–20), and high risk (score: 21+). The 2-year cumulative HCC risk significantly differed among the three risk groups (low risk: 0.94%, moderate risk: 3.25%, and high risk: 9.42%), with log-rank test *p* < 0.001 (Figure 2A). The 5-year cumulative HCC risk significantly differed among the three risk groups (low risk: 1.33%, moderate risk: 4.99%, and high risk: 17.46%), with log-rank test *p* < 0.001.

### 3.6. Kaplan–Meier Survival Curve for Cumulative HCC Risk Determined Using the PAGE-B Predictive Scoring System

The 2-year cumulative HCC risk significantly differed among the three risk groups (low risk: 0.00%, moderate risk: 1.35%, and high risk: 3.79%), with log-rank test *p* < 0.001 (Figure 2B). The 5-year cumulative HCC risk significantly differed among the three risk groups (low risk: 0.00%, moderate risk: 4.13%, and high risk: 7.45%), with log-rank test *p* < 0.001.

## 4. Discussion

Few studies have developed systems for the prediction of HCC risk in treatment-naïve CHB patients receiving NAs that incorporate clinical data from a specific population, and no predictive system is available to date for patients receiving finite periods of NA treatments. The current predictive systems for CHB patients mostly made use of the previous predictive scores (CU-HCC, CAG-HCC, and REACH-B), which were based on the data from CHB patients not receiving NAs to assess HCC risk in patients receiving Nas [23,24,25]. CHB is prevalent in Taiwan [26], and the number of patients with CHB infection receiving NAs is increasing [1]. The accuracy of predictive scores for HCC risk in treatment-naïve CHB patients receiving NAs is unclear. Most importantly, in Taiwan, where National Health Insurance is available, CHB patients are treated with finite periods of antiviral therapies [19]. Our study is the first to assess predictive scores of HCC risk in treatment-naïve CHB patients receiving finite NA treatments. Our findings showed that the 2-year cumulative HCC risk in patients with CHB infection receiving NAs significantly differed among the three risk groups (low risk: 0.94%, moderate risk: 3.25%, and high risk: 9.42%), with log-rank test *p* < 0.001, and that the 5-year cumulative HCC risk in CHB patients receiving NAs also significantly differed among the three risk groups (low risk: 1.33%, intermittent risk: 4.99%, and high risk: 17.46%), with log-rank test *p* < 0.001 (Figure 1). Our study demonstrates the effectiveness of the novel CGMH HCC predictive scoring system, which incorporates several clinical parameters associated with HCC risk (genotype C, relapse after NA withdrawal, male gender, liver cirrhosis, and T1768A HBV mutation). It proves to be a promising tool for predicting the risk of hepatocellular carcinoma in treatment-naïve patients with CHB who receive finite periods of NA treatments. Our study findings align with existing knowledge that HCC risk is higher in the indeterminate phase of CHB compared to the inactive phase. Notably, concurrent hepatic steatosis is independently associated with a lower risk of HCC, whereas the burden of metabolic dysfunction exacerbates HCC risk in untreated CHB patients [27]. Furthermore, the study highlights that antiviral therapy significantly reduces the risk of HCC by 70% among individuals in the indeterminate phase of CHB [28]. These findings hold significant implications for potentially expanding the treatment criteria for CHB [28]. Thus, the CGMH HCC predictive scoring system provides valuable insights into individualized risk assessment and can aid in informing clinical decision-making regarding the management and treatment of CHB patients at risk of developing HCC.

Predicting HCC risk is vital because HCC can still develop in patients receiving NAs. Patients with high-risk scores can be more closely monitored and screened for HCC. Using a stepwise multivariate Cox proportional hazards model, we found that genotype C (aHR: 2.23, score: 2), relapse after NA withdrawal (aHR: 6.96, score: 7), male gender (aHR: 4.19, score: 4), liver cirrhosis (aHR: 11.14, score: 11), and T1768A HBV core promoter mutation (aHR: 3.21, score: 3) were significant independent risk factors for HCC (Table 2). Being male and the presence of liver cirrhosis are widely accepted risk factors for HCC in patients with CHB, regardless of the NA treatment status [4,29,30,31]. Genotype C is a risk factor for HCC among patients with HBV not receiving Nas [5], albeit no study has shown that genotype C is an independent risk factor for HCC in CHB patients receiving NAs. Our study is the first to demonstrate genotype C as an independent risk factor for HCC (aHR: 2.23) in CHB patients, regardless of the NA treatment status. In addition, relapse after NA withdrawal was an independent risk factor for HCC (aHR: 6.96) in treatment-naïve CHB patients receiving finite periods of NA treatments. Few studies have reported relapse after NA withdrawal as an independent risk factor for HCC in patients receiving NAs because the great majority of CHB patients in the world are treated by NA for a lifelong period [24]. In Taiwan, where finite periods of treatments are provided by the National Health Insurance Policy, NA-withdraw-associated hepatitis relapse frequently occurs. In case of clinical and virological relapses, NA can be re-applied as another course of treatment. In our study, relapse after NA treatment was defined as HBV DNA > 2000 IU/mL two times and ALT > ULN. Our study demonstrated that relapse upon NA withdrawal could be an independent risk factor for HCC in CHB patients receiving the finite period treatment strategy. Relapse after NA treatment is most likely caused by discontinuation of NA treatment [32], although virological breakthroughs due to resistant mutants are also possible. The latter is now rarely seen because of the availability of rescue therapies with high resistance barriers. Liver cirrhosis is a well-known independent risk factor for HCC in patients with CHB infection receiving Nas [4,29,30,31]. Liver cirrhosis was incorporated into our predictive scoring system (aHR: 11.14) using a stepwise multivariate Cox proportional hazards model. The HBV T1768A mutation (as one of a quadruple mutation) has been shown to downregulate the expression of p53 and the S-phase kinase-associated protein 2 and promote the cell cycle, leading to HCC development [33]. Although the T1768A mutation alone has not previously been associated with HCC risk in CHB patients receiving NAs, according to our review of the literature, it was a strong risk factor for HCC in our model (aHR: 3.21). In the future, we will conduct preclinical and clinical studies to investigate the mechanisms through which T1768A causes HCC development in patients receiving NAs.

The decision to discontinue NA treatment in high-risk patients has been a topic of debate [20,34]. Current guidelines recommend discontinuing NA treatment in certain patient populations, such as HBeAg-positive patients who achieve specific treatment milestones [15,35]. Some recent guidelines also suggest the possibility of discontinuing long-term NA treatment in HBeAg-negative patients [15,35]. This approach is based on evidence showing that withdrawal of NA treatment in some patients can trigger immune control and potentially lead to HBsAg loss [15,35]. In our study, a subset of patients chose to discontinue NA treatment based on these guidelines. However, it is important to acknowledge that individual preferences and management strategies may vary. Some patients may opt to continue NA treatment indefinitely while waiting for curative therapies for HBV to become available. The decision to discontinue or maintain NA treatment should be made on a case-by-case basis, considering factors such as patient preferences and the guidance of healthcare providers.

Several predictive scoring systems have been developed for assessing the risk of HCC in untreated cohorts of Asian patients with CHB. Among these, the most well-known systems include CU-HCC, CAG-HCC, and REACH-B [23,24,25]. These predictive scoring systems were developed to predict HCC risk in CHB patients not receiving NAs [23,24,25]. Some of these predictive scoring systems have been assessed in Asian patients with CHB infection receiving NAs, specifically entecavir. Wong et al. investigated HCC risk scores in a cohort of 1531 patients with CHB (22% had cirrhosis) receiving entecavir [36]. The 5-year cumulative HCC incidence rates were 12.9% in patients with cirrhosis and 2.1% in patients without cirrhosis [36]. The area under the ROC curve for baseline CU-HCC, CAG-HCC, and REACH-B scores in predicting HCC was 0.80, 0.76, and 0.71, respectively [36]. For the CGMH scores, the area under the ROC curve was 0.86 (Figure 2A). In a large study by Papatheodoridis [37], which included 1666 Caucasian patients with CHB infection receiving entecavir and/or tenofovir, the GAG-HCC, CU-HCC, and REACH-B scores were only associated with HCC development in univariate analysis but not in multivariate analysis. Similarly, in another multicenter randomized controlled trial involving 744 entecavir-treated patients with CHB from 11 European referral centers, the predictive ability of CU-HCC, CAG-HCC, and REACH-B scores for HCC was found to be low [38]. It is worth noting that these studies primarily focused on patients receiving only one or two types of NAs, specifically entecavir and/or tenofovir [37,38], and did not consider patients receiving various NAs. Moreover, these predictive scoring systems did not incorporate genetic testing for HBV point mutations [23,24,25,36,37,38]. Our study is the first to investigate the association of HBV point mutations with HCC risk in patients with CHB infection receiving different NAs. Furthermore, our study had a longer follow-up time than the other studies.

To improve AUC scores in our CGMH HCC predictive model, we took several steps. We included relevant variables such as genotype C, NA-withdraw-related hepatitis relapse, male gender, liver cirrhosis, and T1768A HBV core promoter mutation. We addressed collinearity through stepwise selection and minimized redundancy. With a cohort of 790 patients, we had a sufficient sample size to capture patterns and improve AUC scores. We considered non-linear relationships and complex interactions for accurate HCC risk assessment. We balanced model complexity to prevent overfitting or underfitting and optimized generalization performance. Additionally, we analyzed HCC risk among three risk groups to handle data imbalance. By considering these factors—relevant predictors, collinearity, sample size, non-linearity, overfitting or underfitting, and data imbalance—we aimed to enhance AUC scores in our CGMH HCC predictive model. We also utilized appropriate modeling techniques and optimized model parameters.

The generalizability of the aforementioned predictive scoring systems and that of the CGMH predictive scoring system in this study to non-Asian patients is unclear. A recent study by Papatheodoridis et al. [22] aimed to develop and validate an HCC risk scoring system for Caucasian patients with CHB who were undergoing antiviral therapy. The study included 1815 adult Caucasian patients who had received entecavir or tenofovir for a minimum of 12 months. The researchers successfully developed a straightforward and reliable HCC risk scoring system known as PAGE-B. This scoring system, which incorporates platelet count, age, and gender, accurately predicts the risk of HCC development within 5 years of therapy. However, their study did not include genetic tests or any variation in NA use. The areas under the ROC curves for HCC prediction were 0.70 for the baseline PAGE-B scores and 0.86 for our scores (Figure 1). By using complete and accurate data, we established a reliable predictive scoring system that exhibited significant differences in HCC risk among low-risk, moderate-risk, and high-risk groups. By contrast, the PAGE-B scoring system exhibited significant differences in HCC risk among treatment-naïve Asian patients with CHB infection receiving NAs, although the curves of cumulative HCC risk were closer than ours (Figure 2). Our study aimed to develop a predictive scoring system for HCC risk in treatment-naïve CHB patients receiving finite periods of NA treatments. The CGMH HCC predictive scoring system, incorporating clinical parameters like genotype C, relapse after NA withdrawal, male gender, liver cirrhosis, and T1768A HBV mutation, showed promise in predicting HCC risk. Comparing our system to the PAGE-B scoring system developed for Caucasian patients, our CGMH scores demonstrated higher accuracy with a higher AUC, indicating better sensitivity and specificity in predicting HCC risk (Figure 1). Moreover, our system exhibited a wider range between different risk groups, indicating better differentiation of HCC risk among the population (Figure 2). However, the generalizability of our system and others to non-Asian patients remains uncertain.

In our CGMH predictive model, we have identified and incorporated several risk factors associated with hepatocellular carcinoma (HCC). These risk factors include genotype C, NA-withdraw-related hepatitis relapse, male gender, liver cirrhosis, and the T1768A HBV core promoter mutation. By considering these risk factors, we can assess the probability of developing HCC in individuals. The dramatic difference between the lines suggests that these individuals with higher risk scores exhibit a combination of multiple risk factors, leading to a significantly elevated risk of HCC compared to individuals with lower scores. The specific components/risk factors contributing to this dramatic difference can be attributed to the cumulative effect and interaction of the identified risk factors in the model. Understanding the components/risk factors in the yellow line for individuals with scores > 21 in A and >18 in B is crucial for providing tailored advice to colleagues (Figure 2). By identifying and addressing these specific risk factors, healthcare professionals can offer targeted interventions, closer monitoring, and appropriate management strategies to individuals at higher risk, aiming to reduce the incidence or progression of HCC.

Our study has several limitations that should be considered when interpreting the findings. First, the retrospective nature of the study design introduces inherent limitations, such as potential selection bias and incomplete data collection. Although efforts were made to minimize these biases, there may still be residual confounding factors that could influence the observed outcomes. Second, the generalizability of our results may be limited to patients receiving finite periods of antiviral treatment for CHB. The study population was derived from a single center, which may not fully represent the broader CHB patient population. Additionally, the small number of patients receiving interferon-based treatment poses challenges in conducting detailed subgroup analyses within this group. The limited sample size may result in unstable estimates and reduced statistical power to detect meaningful differences. Third, it is essential to acknowledge the inherent limitations of the predictive scoring system for HCC risk in treatment-naïve CHB patients. Although our study demonstrated significant differences in HCC incidence among the risk groups using the scoring system, we acknowledge that the current sample size of our dataset is insufficient to randomly select an independent validation sample set for validating the CGMH HCC predictive scores. Therefore, to assess the generalizability and accuracy of the scoring system, it is crucial to conduct validation in larger, independent cohorts in the future. This validation process will provide a more comprehensive evaluation of the scoring system’s performance and significantly enhance its applicability in clinical settings.

## 5. Conclusions

The novel CGMH HCC predictive scoring system, which incorporates HCC risk-related clinical parameters (genotype C, relapse after NA withdrawal, male gender, liver cirrhosis, and T1768A HBV mutation), is a promising tool for predicting HCC risk in treatment-naïve CHB patients receiving finite periods of NA treatments.

## Figures and Tables

**Table 1:** Patient Characteristics.

Characteristics	Patients with CHB Receiving NAs without HCC	Patients with CHB Receiving NAs with HCC	*p* Value		
	N = 757	N = 33			
	N	%	N	%	
Age					
Mean ± SD	48.40 ± 12.84	53.46 ± 8.34	0.025		
Median (IQR)	48.94 (39.38, 57.03)	53.61 (45.79, 58.36)	0.015		
Gender					0.028
Female	226	29.9%	4	12.1%	
Male	531	70.2%	29	87.9%	
Laboratory data					
Alpha-fetoprotein (ng/mL)					
Mean ± SD	24.15 ± 83.76	11.14 ± 18.26	0.373		
Median (IQR)	4.30 (3.00, 9.20)	4.00 (3.00, 7.00)	0.966		
Aspartate aminotransferase (U/L)					
Mean ± SD	175.51 ± 306.87	74.36 ± 68.01	0.059		
Median (IQR)	72.00 (44.00, 148.00)	49.00 (39.00, 80.00)	0.016		
Alanine aminotransferase (U/L)					
Mean ± SD	245.98 ± 393.09	81.45 ± 76.87	0.017		
Median (IQR)	115.00 (60.00, 232.00)	50.00 (37.00, 103.00)	<.0001		
Total Bilirubin (mg/dL)					
Mean ± SD	1.50 ± 2.98		1.22 ± 2.12		0.593
Median (IQR)	0.80 (0.50, 1.10)	0.70 (0.50, 1.20)	0.603		
International normalized ratio					
Mean ± SD	1.18 ± 0.81		1.14 ± 0.15		0.761
Median (IQR)	1.00 (1.00, 1.10)	1.10 (1.00, 1.20)	0.059		
Platelet (10^3^/μL)					
Mean ± SD	178.16 ± 72.86	141.64 ± 50.73	0.005		
Median (IQR)	174.00 (140.00, 210.0)	144.00 (117.00, 165.0)	0.001		
HBV DNA titer					
HBV DNA (copies/mL)					
Mean ± SD	917.82 ± 5672.48	166.67 ± 799.77	0.448		
Median (IQR)	25.30 (1.51, 640.20)	4.09 (1.24, 23.63)	0.018		
_log_HBV DNA					
Mean ± SD	7.30 ± 1.61		6.77 ± 1.00		0.058
Median (IQR)	7.40 (6.18, 8.81)	6.61 (6.09, 7.37)	0.018		
Relapse after NA withdraw					0.002
No	233	30.8%	2	6.1%	
Yes	524	69.2%	31	93.9%	
Courses of NA treatment					0.655
1	575	76.0%	27	81.8%	
2	149	19.7%	6	18.2%	
3	27	3.6%	0	0.0%	
4	6	0.8%	0	0.0%	
Antiviral treatment strategies					
Peginterferon in combination with entecavir					0.240
No	722	95.4%	30	90.9%	
Yes	35	4.6%	3	9.1%	
Entecavir monotherapy followed by NA switching or addition					0.065
No	353	46.6%	10	30.3%	
Yes	404	53.4%	23	69.7%	
Entecavir monotherapy					0.018
No	318	42.0%	7	21.2%	
Yes	439	58.0%	26	78.8%	
Tenofovir monotherapy switching to or addition of other NAs					0.856
No	657	86.8%	29	87.9%	
Yes	100	13.2%	4	12.1%	
Initial lamivudine or telbivudine therapy					0.042
No	602	79.5%	31	93.9%	
Yes	155	20.5%	2	6.1%	
Peginterferon monotherapy					0.275
No	694	91.7%	32	97.0%	
Yes	63	8.3%	1	3.0%	
HBeAg					0.418
Negative	499	65.9%	24	72.7%	
Positive	258	34.1%	9	27.3%	
Anti-HBe					0.044
Negative	240	31.7%	5	15.2%	
Positive	517	68.3%	28	84.9%	
Liver Cirrhosis					<.0001
No	591	78.1%	7	21.2%	
Yes	166	21.9%	26	78.8%	
HBV Genotype					
Genotype B					0.004
No	177	23.4%	15	45.5%	
Yes	580	76.6%	18	54.6%	
Genotype C					0.008
No	586	77.4%	19	57.6%	
Yes	171	22.6%	14	42.4%	
HBV viral point mutation					
G1719T					0.034
No	712	94.1%	28	84.9%	
Yes	45	5.9%	5	15.2%	
G172A/C					0.506
No	747	98.7%	33	100.0%	
Yes	10	1.3%	0	0.0%	
C1730G					0.005
No	575	76.0%	18	54.6%	
Yes	182	24.0%	15	45.5%	
A1752G					0.519
No	486	64.2%	23	69.7%	
Yes	271	35.8%	10	30.3%	
T1753C/A					0.713
No	659	87.1%	28	84.9%	
Yes	98	13.0%	5	15.2%	
A1762T					0.003
No	428	56.5%	10	30.3%	
Yes	329	43.5%	23	69.7%	
G1764A					0.001
No	413	54.6%	8	24.2%	
Yes	344	45.4%	25	75.8%	
C1766T					0.038
No	711	93.9%	28	84.9%	
Yes	46	6.1%	5	15.2%	
T1768A					0.022
No	715	94.5%	28	84.9%	
Yes	42	5.6%	5	15.2%	
C1799G					0.012
No	176	23.3%	14	42.4%	
Yes	581	76.8%	19	57.6%	
G1896A					0.829
No	243	32.1%	10	30.3%	
Yes	514	67.9%	23	69.7%	
G1899A					0.574
No	604	79.8%	25	75.8%	
Yes	153	20.2%	8	24.2%	

Abbreviations: HCC, hepatocellular carcinoma; HBV, hepatitis B virus; CHB, chronic hepatitis B virus; NA, nucleos(t)ide analogue; HBsAg, HBV surface antigen; HBeAg, HBe antigen; anti-HBe, antibody against HBe; AST, aspartate transaminase; ALT, alanine transaminase; SD, standard deviation; IQR, interquartile range; ng, nanogram; mL, milliliter.

**Table 2:** Stepwise Multivariate Cox Proportional Hazards Model for Hepatocellular Carcinoma Risk in Treatment-Naïve CHB Patients Receiving Finite NA Treatments.

Factor	aHR *	95% CI		*p* Value	Assigned Points
Genotype C	2.23	1.09	4.55	0.029	2
Relapse after NA withdraw	6.96	1.64	29.62	0.009	7
Male	4.19	1.43	12.33	0.009	4
Liver Cirrhosis	11.14	4.79	25.92	<0.0001	11
T1768A	3.21	1.19	8.69	0.022	3

Abbreviations: aHR, adjusted hazard ratio; CI, confidence interval; NA, nucleos(t)ide analogue. * All variables in Table 1 were used in multivariate analysis.

**Table 3:** Hepatocellular Carcinoma Risk Assessment Using CGMH Predictive Scoring System for Treatment-Naïve CHB Patients Receiving Finite NA Treatments.

CGMH Predictive Scoring System	Number of Patients without HCC	Number of Patients with HCC	HCC Incidence
0	36	0	0.00%
2	13	0	0.00%
3	5	0	0.00%
4	104	0	0.00%
5	1	0	0.00%
6	32	0	0.00%
7	93	1	1.06%
9	29	1	3.33%
11	227	3	1.30%
12	7	0	0.00%
13	39	2	4.88%
14	4	0	0.00%
15	19	1	5.00%
16	8	0	0.00%
17	7	0	0.00%
18	23	1	4.17%
20	16	1	5.88%
21	3	0	0.00%
22	70	12	14.63%
23	2	1	33.33%
24	16	7	30.43%
25	2	1	33.33%
27	1	2	66.67%
Total	757	33	4.17%

Abbreviations: CGMH, Chang Gung Memorial Hospital; HCC, Hepatocellular carcinoma; NA, nucleos(t)ide analogue.

**Table 4:** Hepatocellular Carcinoma Risk Assessment Using CGMH Predictive Scoring System.

CGMH Predictive Scoring	Group	HCC Risk	
Score		No HCC, N (%)	HCC, N (%)
0–12	Low risk	547 (99.09%)	5 (0.91%)
13–20	Moderate risk	116 (95.87%)	5 (4.13%)
≥21	High risk	94 (80.34%)	23 (19.66%)

Abbreviations: CGMH, Chang Gung Memorial Hospital; HCC, Hepatocellular carcinoma; N, number.

## Data Availability

The datasets supporting the study conclusions are included within this manuscript and Supplementary Materials.

## Supplementary Materials

The following supporting information can be downloaded at: https://www.mdpi.com/article/10.3390/cancers15133343/s1, Table S1. Hepatocellular Carcinoma Risk Assessment Using PAGE-B Predictive Scoring System.

## Abbreviations

HCC, hepatocellular carcinoma; HBV, hepatitis B virus; CHB, chronic hepatitis B virus; NA, nucleos(t)ide analogue; HBsAg, HBV surface antigen; HBeAg, HBe antigen; anti-HBe, antibody against HBe; AST, aspartate transaminase; ALT, alanine transaminase; BCP, basal core promoter; HR, hazard ratio; aHR, adjusted hazard ratio (aHR); CI, confidence interval.
