# Supplementary material for: Predicting Hepatocellular Carcinoma Risk in Chronic Hepatitis B Patients Receiving Finite Periods of Antiviral Therapy
Source: Cancers (Basel). 2023 Jun 25;15(13):3343. doi: 10.3390/cancers15133343 (PMC10340409; doi:10.3390/cancers15133343)
Supplement: Supplementary file 1 [file cancers-15-03343-s001.zip › cancers-2448900-supplementary.pdf]

cancers

MDPI

Supplementary Material

Predicting Hepatocellular Carcinoma Risk in Chronic Hepatitis B Patients Receiving Finite Periods of Antiviral Therapy

Chih-Lang Lin, MD[1,2,3,†]; Szu-Yuan Wu, MD, MPH, PhD.[6,7,8,9,10,11,12,†,*]; Ming-Wei Lai, MD, PhD[3,4,5]; Chao-Wei Hsu, MD[3,4]; Wan-Ming Chen, PhD[4,5]; An-Tzu Jao, BS[6]; Cheng-Hung Chien, MD[1,2,3]; Ching-Chih Hu, MD, PhD.[1,3]; Rong-Nan Chien, MD[3,4,*]; Chau-Ting Yeh, MD, PhD.[3,4,*]

Supplemental Table S1. Hepatocellular Carcinoma Risk Assessment Using PAGE-B Predictive Scoring System

CGMH Predictive Scoring	Group	HCC Risk	
**Score**		**No HCC, N (%)**	**HCC, N (%)**
**0–9**	**Low risk**	159 (100.00%)	0 (0.00%)
**10–17**	**Moderate risk**	398 (95.90%)	17 (4.10%)
**≥18**	**High risk**	200 (92.59%)	16 (7.41%)

Abbreviations: CGMH, Chang Gung Memorial Hospital; HCC, Hepatocellular carcinoma; N, number.
